# Reproducing and quantitatively validating a biologically-constrained point-neuron model of CA1 pyramidal cells

**DOI:** 10.3389/fnint.2022.1041423

**Published:** 2022-11-08

**Authors:** Shailesh Appukuttan, Andrew P. Davison

**Affiliations:** Université Paris-Saclay, CNRS, Institut des Neurosciences Paris-Saclay, Saclay, France

**Keywords:** reproducibility, validation, verification, testing, SciUnit, EBRAINS Model Catalog

## Abstract

We have attempted to reproduce a biologically-constrained point-neuron model of CA1 pyramidal cells. The original models, developed for the Brian simulator, captured the frequency-current profiles of both strongly and weakly adapting cells. As part of the present study, we reproduced the model for different simulators, namely Brian2 and NEURON. The reproductions were attempted independent of the original Brian implementation, relying solely on the published article. The different implementations were quantitatively validated, to evaluate how well they mirror the original model. Additional tests were developed and packaged into a test suite, that helped further characterize and compare various aspects of these models, beyond the scope of the original study. Overall, we were able to reproduce the core features of the model, but observed certain unaccountable discrepancies. We demonstrate an approach for undertaking these evaluations, using the SciUnit framework, that allows for such quantitative validations of scientific models, to verify their accurate replication and/or reproductions. All resources employed and developed in our study have been publicly shared *via* the EBRAINS Live Papers platform.

## 1. Introduction

Computational investigations, by means of developing models, are today an intrinsic part of research in neuroscience. Modeling can be undertaken at various levels. The study presented here focuses on single cell models. Such models are typically of two broad types: biologically realistic spatial models or point-neuron models. Spatial models take into account the three-dimensional morphology, modeling the various parts such as soma, axon and dendrites. The degree of spatial detail in these models can vary considerably. The point-neuron models, on the other hand, condense the entire neuron to a single point in space, and thus have no spatial profile. The choice of models, whether to employ a spatial model or a point-neuron model, is determined largely by the objective of the study, as well as the computational feasibility to undertake such studies. For example, to study behavior such as dendritic integration, it is necessary to have the dendrites modeled explicitly, and therefore spatial models would be an appropriate choice (Katz et al., 2009). But if the focus is solely on mimicking various patterns of spiking activity, then a point-neuron model could suffice (Izhikevich, 2003). Spatial models are inherently more complex than point-neuron models, and therefore demand more computational power. At the level of single cell simulations, these differences are often easily manageable with present day computing resources. But when moving to the network level, consisting of a large population of individual neurons, the computational load escalates rapidly and can become prohibitively large to employ detailed spatial models. For this reason, point-neuron models are often the preferred choice for developing neuronal network models (Potjans and Diesmann, 2014).

The domain of computational modeling currently involves a host of different simulators. Some examples are NEURON (Hines and Carnevale, 1997), NEST (Eppler et al., 2009), *Brian2* (Stimberg et al., 2019), Arbor (Abi Akar et al., 2019), and Moose (Ray et al., 2008). These simulators typically have their own model description languages, and converting from one format to another is often non-trivial. This can lead to fragmentation of research efforts, wherein models developed by one team cannot be adopted and extended upon by another team, if they intend to employ different simulators. This makes it essential to ensure that publications associated with modeling studies describe in sufficient detail the steps underlying the development of the model, as well as the protocols underlying the reported simulation findings. This would empower any researcher to reproduce the same model on a different platform, thereby enabling both validation of the original model, and the continuation of the same work.

It is equally important to ensure that any replicated and/or reproduced models are closely compared and validated with the original model. Typically, when validating models, be it comparing model outcomes to experimental data, or comparing the responses of multiple models, the assessment of match is undertaken by means of visual likeness. Needless to say, such comparisons lack scientific rigor, and can often be subjective and prone to bias. A more appropriate evaluation should quantitatively assess the closeness of the match using a suitable metric. Certain minor discrepancies can often be attributed to simulator-specific differences, but more evident errors are likely to indicate faults in the reproduction. In some cases, this can even possibly help identify flaws in the simulator itself. The value in reproducing published models is therefore multi-fold, as reflected in the aims of this research topic on “Reproducibility in Neuroscience”, and integral to the scientific progress of the field.

Here, we attempted to reproduce one such modeling study involving the development of biologically constrained point-neuron models of CA1 pyramidal cells (Ferguson et al., 2014). Ferguson et al. (2014) developed single cell models of both strongly and weakly adapting CA1 pyramidal cells, using the Brian simulator (Goodman and Brette, 2009), which capture the frequency-current profile of the cells, and also exhibit expected behavior such as rebound spiking. Here, we reproduce these models using different simulators, namely, Brian2 and NEURON. These replications were attempted solely based on the details provided in the published article, but the lack of information on certain parameters required borrowing these from the source code published by the authors on ModelDB (Accession#: 182515) (Hines et al., 2004). Subsequently, we compared the behavior of these reproduced models against the original published Brian (different from Brian2) implementation. These comparisons are conducted quantitatively using SciUnit (Omar et al., 2014), and the EBRAINS Model Validation Framework (Appukuttan et al., 2022b). A test suite, named *eFELunit*, was developed in Python as a distributable package, containing several validation tests in addition to those described in the original study. This allows for a more comprehensive evaluation of the various aspects of the different implementations of the model.

In brief, our objectives with this study were two-fold: (i) to verify if the description of the model provided in the published study is sufficient to accurately reproduce the original findings and (ii) to demonstrate a generalized approach for quantitatively validating the reproducibility of published models.

## 2. Methods

An overview of the original model, and its reproductions is provided below. All the resources employed and/or produced as part of this study, including all model source code, analyses code, output figures and files, are publicly available *via* the associated EBRAINS Live Paper (Appukuttan et al., 2022a); see the Data Availability Statement. Detailed instructions for setting up the simulation environments are also provided.

### 2.1. Original model

The model equations are a modified version of those for the Izhikevich model (Ferguson et al., 2014). The model employs a different *k* parameter below and above the spike threshold (see Equation 4). The modifications allow the model to better capture several biophysical properties of CA1 pyramidal cells. The equations governing the model are:


(1) $$\begin{array}{l} C_{\text{m}}\frac{dV}{dt}=k\left(V-v_{\text{r}}\right)\left(V-v_{\text{t}}\right)-u+I_{\text{applied}}+I_{\text{shift}} \end{array}$$

(2) $$\begin{array}{l} \frac{du}{dt}=a\left[b\left(V-v_{\text{r}}\right)-u\right] \end{array}$$

(3) $$\begin{array}{l} \text{if }V\geq v_{\text{peak}},\text{then }v\leftarrow c,u\leftarrow u+d \end{array}$$

(4) $$\begin{array}{l} \text{where }k=k_{\text{low}}\;if\;V\leq v_{\text{t}};k=k_{\text{high}}\text{ if }V>v_{\text{t}} \end{array}$$

where *V* (in mV) is the membrane potential, *u* (in pA) is a slow recovery current, *C*_m_ (in pF) is the membrane capacitance, *v*_r_ (in mV) is the resting membrane potential, *v*_t_ (in mV) is the spike threshold potential, *v*_peak_ (in mV) is the spike cut-off value, *I*_applied_ (in pA) is the stimulus current, *I*_shift_ (in pA) is a current for laterally shifting the f-I curve, *a* (in ms^-1^) is the recovery time constant, *b* (in nS) dictates the sensitivity of the recovery current to sub-threshold changes in potential, *c* (in mV) is the voltage reset value following a spike, *d* (in pA) describes the after-spike reset of the recovery current, and *k*, *k*_low_, *k*_high_ are scaling factors.

The values employed for the above parameters are listed in Table 1. Based on the three sets of parameters, Ferguson et al. (2014) obtained three model variants: strongly adapting pyramidal cell model, weakly adapting pyramidal cell model #1 and weakly adapting pyramidal cell model #2. We shall refer to these three variants as *Pyr*_*Strong*, *Pyr*_*Weak1*, and *Pyr*_*Weak2*, respectively.

**Table 1:** Parameters employed for the different model variants corresponding to the terms in the equations.

**Parameter**	**Model**		
	**Strongly Adapting**	**Weakly Adapting**	
	**Pyr_Strong**	**Pyr_Weak1**	**Pyr_Weak2**
*v*_r_	−61.8 mV	−61.8 mV	−61.8 mV
*v*_t_	−57.0 mV	−57.0 mV	−57.0 mV
*c*	−65.8 mV	−65.8 mV	−65.8 mV
*v*_peak_	22.6 mV	22.6 mV	22.6 mV
*k*_high_	3.3 nS/mV	3.3 nS/mV	3.3 nS/mV
*C*_m_	115 pF	300 pF	300 pF
*a*	0.0012 ms^-1^	0.001 ms^-1^	0.00008 ms^-1^
*b*	3 nS	3 nS	3 nS
*d*	10 pA	5 pA	5 pA
*k*_low_	0.1 nS/mV	0.5 nS/mV	0.5 nS/mV
*I*_shift_	0 pA	−45 pA	−45 pA

Note that the parameters *C*_m_, *a*, *d*, *k*_low_, *I*_shift_ differ between the strongly adapting and weakly adapting models. Only the parameter *a* varies between the two weakly adapting models.

The published study is available online in two versions; the second version addresses comments provided by the reviewers during the open-review process. We shall refer to this version of the publication in our discussions.

### 2.2. Model reproductions

We implemented the model for the *Brian2* and NEURON simulators based on the description of the models provided in the published article. We introduced a small variation in the model equations that allowed us to specify the start ($t_{\text{start}}^{\text{stim}}$) and stop time ($t_{\text{stop}}^{\text{stim}}$) of the stimulus, thereby allowing us to better control the simulations. This was introduced by updating Equation (1) as follows:


(5) $$\begin{array}{l} C_{\text{m}}\frac{dV}{dt}=k\left(V-v_{\text{r}}\right)\left(V-v_{\text{t}}\right)-u+I_{\text{ext}}+I_{\text{shift}} \end{array}$$

where


(6) $$\begin{array}{l} I_{\text{ext}}=I_{\text{applied}}\;if\;t\geq t_{\text{start}}^{\text{stim}}\;and\;t<t_{\text{stop}}^{\text{stim}},otherwiseI_{\text{ext}}=0 \end{array}$$

As the underlying equations remained the same for all the model variants, which only varied in the values of the parameter set, we developed a single class-based template for the pyramidal cell. The three model variants could be instantiated from these templates, by applying the required parameter set. This approach was largely motivated with an eye to minimizing variations between the implementations of the three variants, and to promote code reuse. Furthermore, the implementations were designed to be compatible with SciUnit, which offers a framework for validating scientific models. We will discuss this in more detail in the following section. For the purposes of discussion, let us refer to these two sets of models as *Brian2* and *NEURON*, each consisting of the three variants. The differential equations in the *NEURON* models' NMODL files were solved using the *euler* integration method, to correspond to the original study.

Ferguson et al. (2014) developed the original model using the Brian simulator. The source code for the model is available on ModelDB (Accession#: 182515). We wanted to avoid any major changes to the original source code, to ensure the model was used as provided (except for fixing some minor syntactical errors in context to running simulations on the Brian simulator). We therefore created an additional version of the Brian model, designed as a class-based template, similar to the Brian2 and NEURON implementations, and also incorporated the SciUnit interface. We shall refer to these two sets of models as *Original* and *Brian1*. Both these models employ the Brian simulator, and if the modified *Brian1* implementation is a faithful reproduction of the *Original* implementation, they should produce identical responses. All the models are registered in the EBRAINS Model Catalog, thereby providing access to all associated metadata and validation results.

The value of the initial resting potential for the models was not specified in the published article, and the published source code picks this randomly from a uniform distribution between −55.0 and −75.0 mV. For purposes of reproducibility and consistency, we deemed it essential to maintain a constant initial membrane potential across all simulation runs. We therefore used −65.0 mV for this parameter, corresponding to the mean value. For certain simulations, we explored the effect of altered initial membrane potential. Where applicable, this has been clearly indicated in the text. The simulation time step was set to 0.02 ms for all runs, in accordance with that specified in the published source code.

### 2.3. Validation tests

**Figure 1:**
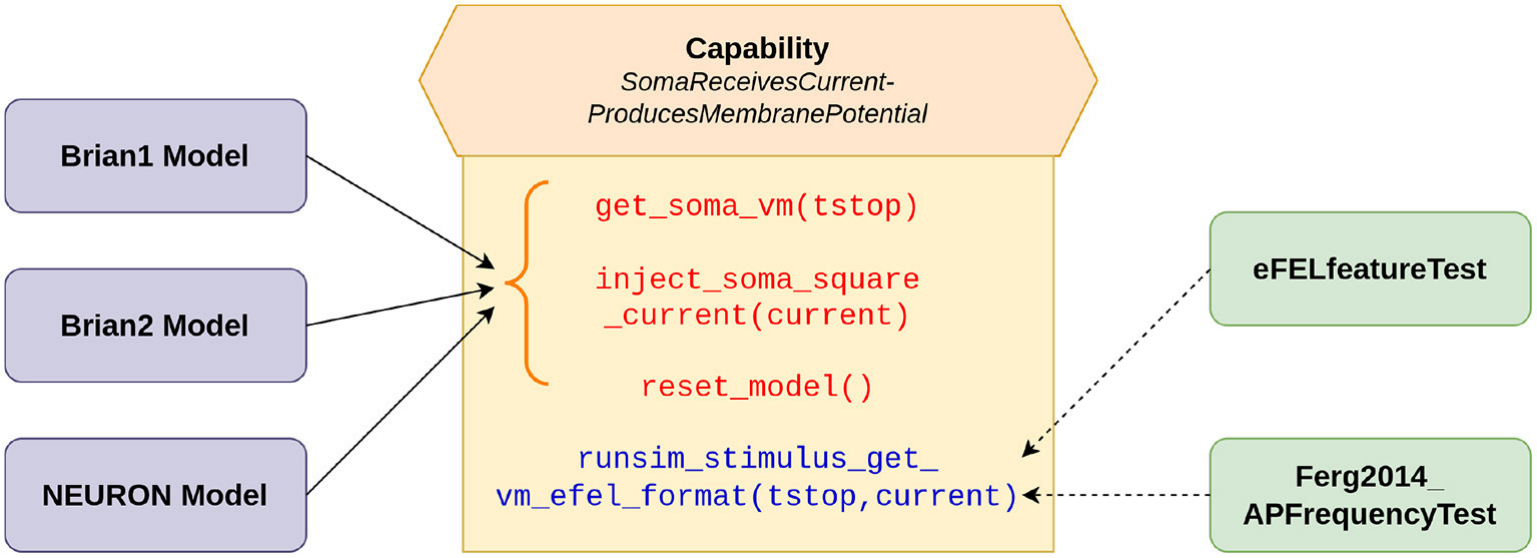
SciUnit based workflow of running validation tests. The capability acts as an interface between the models and tests, and defines the functionalities demanded from the models. The solid arrows indicate the functions (in red) that are to be implemented by our models; these functions represent more granular functionalities. The function (in blue) combines these more basic functions, to provide a more comprehensive workflow, and can be directly utilized (shown by dashed arrows) by the tests to evaluate the model.

The validation tests were developed using the SciUnit framework (Omar et al., 2014). SciUnit provides a framework for developing model-agnostic tests. Tests can be written to be completely independent of the internal details of the models and their implementations. This allows a test to be written once, and used across multiple models. SciUnit achieves this through the concept of “Capabilities”, which represent clearly defined interfaces through which tests can communicate with models. Figure 1 illustrates the relationship between models and tests as applicable for the current study. The tests specify the functionalities that they require from the models to undertake the required validation. These requirements are defined in the capability named *SomaReceivesCurrentProducesMembranePotential* and include abilities such as to: (i) record the membrane potential from soma: *get*_*soma*_*vm*(*tstop*), (ii) inject stimulus as a square pulse of current into the soma: *inject*_*soma*_*square*_*current*(*current*), (iii) reset the model to its initial state: *reset*_*model*(). Additionally, the capability self-implements the function *runsim*_*stimulus*_*get*_*vm*_*efel*_*format*(*tstop, current*), which makes use of the other functions to bring the model to the initial state, inject the required stimulus, and to simulate and record the membrane potential for the specified duration. The output is produced in the format required by the eFEL library (described below). This is used by the tests to perform the required evaluations. The models, wrapped with a SciUnit interface, are expected to fulfill the requirements defined in the capability to be eligible to undertake these validation tests. The onus, therefore, is on the model developers to satisfy these requirements as part of the model source code. Our *Brian1*, *Brian2*, and *NEURON* models meet these requirements. Gerkin and Omar (2013) provides a more detailed description of the development of such model-agnostic tests using the SciUnit framework.

To verify the integrity of the reproductions, we carried out two levels of testing. The first level consisted of reproducing the results from the original publication. This included comparing features such as the initial and final spiking frequencies for a range of strengths of injected current and comparing the responses for a depolarizing and a hyperpolarizing step of current. The second level consisted of testing additional parameters associated with spike shape and events, as well as testing the passive properties *via* hyperpolarizing stimuli. In all cases, the biophysical features were extracted from the simulation recordings using the Electrophys Feature Extraction Library (eFEL) (BlueBrainProject, 2015), and all extracted values were rounded-off to two decimal places. This ensured a uniform and reliable approach to evaluating the features of interest across all the models. Each of the tests involved simulations of the model over a range of stimulus strengths. The goodness-of-fit between the simulated data and the target reference data was evaluated using the root mean squared error (RMSE), which has the same units as the parameter being evaluated. All validation tests were registered in the EBRAINS Model Catalog, together with their target observation data, and packaged as a Python library named *eFELunit*.

All four sets of models (*Original*, *Brian1*, *Brian2*, *NEURON*) were tested in the first level of validations. The eFEL features *inv*_*first*_*ISI* (inverse of first inter-spike interval) and *inv*_*last*_*ISI* (inverse of last inter-spike interval) were used to evaluate the initial and final spiking frequencies for each of the model variants (*Pyr*_*Strong*, *Pyr*_*Weak1*, and *Pyr*_*Weak2*). A small adjustment was incorporated in the feature evaluation, whereby if only a single spike was recorded, then the initial and final frequency was set to 1 Hz. This was in accordance with the protocol adopted in the original study. These tests were packaged into a test class named *Ferg2014*_*APFrequencyTest*, as indicated in Figure 1. As the *Original* set of models were not implemented with the SciUnit framework, their tests have to be run separately, but the features were still extracted using the eFEL library. The outputs of the *Original* models were saved as the target reference data for the other sets of models (*Brian1*, *Brian2*, *NEURON*).

In the second level of testing, we only compared the *Brian1*, *Brian2*, and *NEURON* models, with the response of the *Brian1* models set as the target reference data. All models in this case were SciUnit compatible, and the testing could therefore be better streamlined. Here, we evaluated features such as the spike amplitudes (first, second, and last), widths (first, second, and last), time to spike (first, second, and last), the total spike count, and the current-voltage relationship for hyperpolarizing stimuli. These were packaged into a separate test class named *eFELfeatureTest*.

### 2.4. Simulation environments

Development of the Brian simulator has been discontinued, and it only works with Python 2, which itself is no longer supported. Most latest releases of packages are no longer compatible with Python 2, and therefore it was not possible to run all the simulations for the original model and its reproductions, on *Brian2* and NEURON, under a single environment.

We therefore created two virtual environments—one for Python 2 and the other for Python 3. The Python 2 environment was used to run the simulations of the original Brian model (*Original*), and also its SciUnit-wrapped version (*Brian1*). The Python 3 environment was used to run the simulations of the *Brian2* and NEURON versions of the model (i.e., *Brian2* and *NEURON*). Additionally, all the analysis code was run in the Python 3 environment. Instructions for creating both virtual environments have been provided in the associated EBRAINS Live Paper. It also includes details such as versions of all the packages employed in the study.

## 3. Results

Two levels of testing were undertaken. In the first level, we compare the models based on the features evaluated in the original study. The behavior of the *Brian1* model is expected to be exactly identical to the *Original* model, given that both are developed for the same simulator, Brian. Minor simulator-related deviations are expected for the other simulator implementations, and these differences are evaluated and quantified. In the second level, we compare the SciUnit-compatible model implementations with respect to additional aspects of their responses.

### 3.1. Validations: Level I

In the original study, the models are largely characterized and compared to the experimental data *via* their f-I curves and visual examination of the model's response to a depolarizing and hyperpolarizing step of current. We follow the same process, but comparing the reproductions against the *Original* model, rather than the experimental data. This is in line with the objective of the current study, which is to verify the reproducibility of the published model. We aim to perform this comparison not just qualitatively, but quantitatively as well, to obtain a “score” that indicates the closeness of the match.

**Figure 2:**
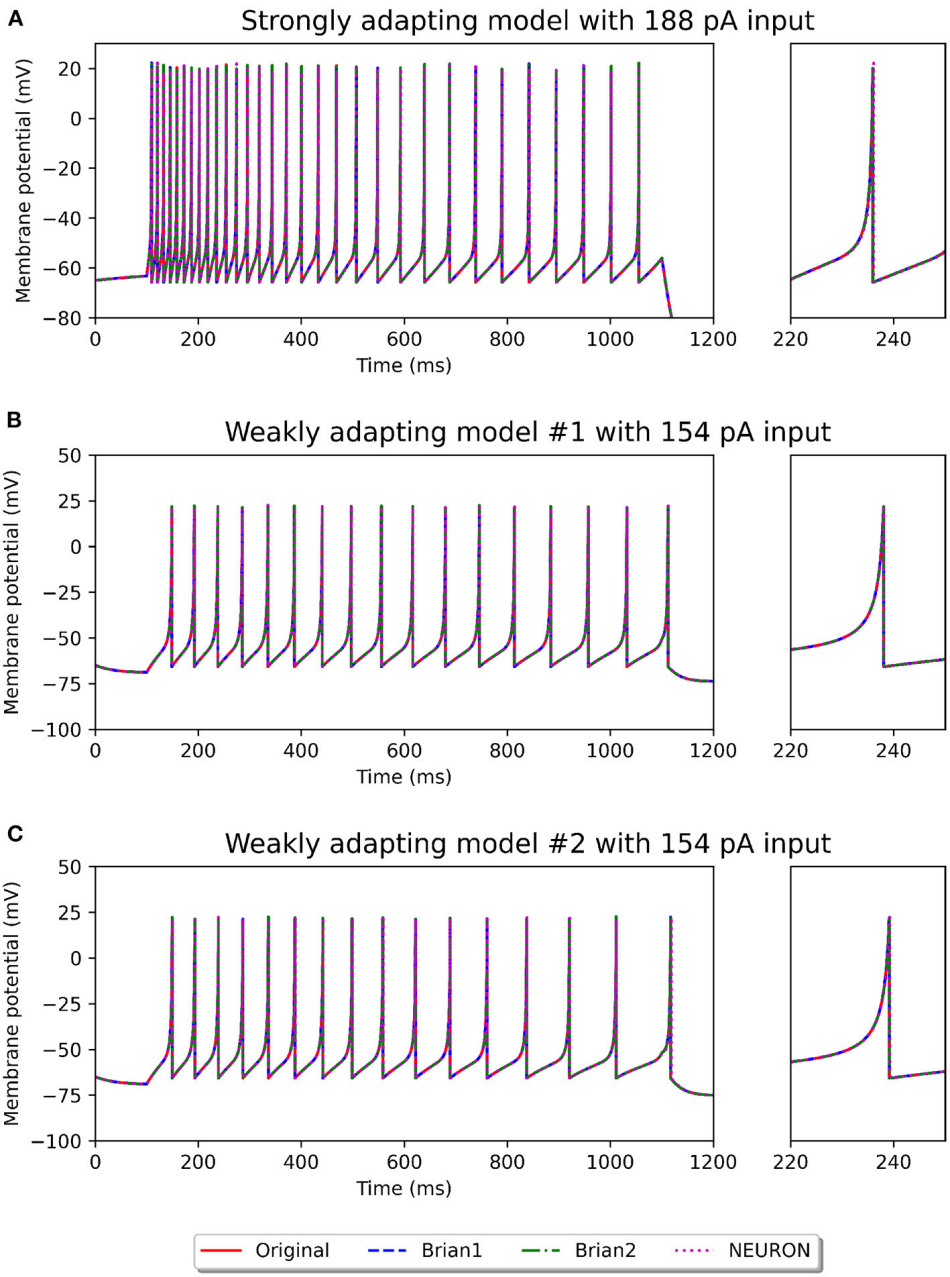
Spiking activity observed in the different model variants following a depolarizing stimulus. The stimulus strengths were chosen based on the examples in the original study: 188 pA stimulus for the strongly adapting models **(A)**, and 154 pA stimulus for the weakly adapting models **(B,C)**. The differences in adaptation is easily noticeable. The smaller panels on the right provide a closer view of one of the spikes in the recording.

**Figure 3:**
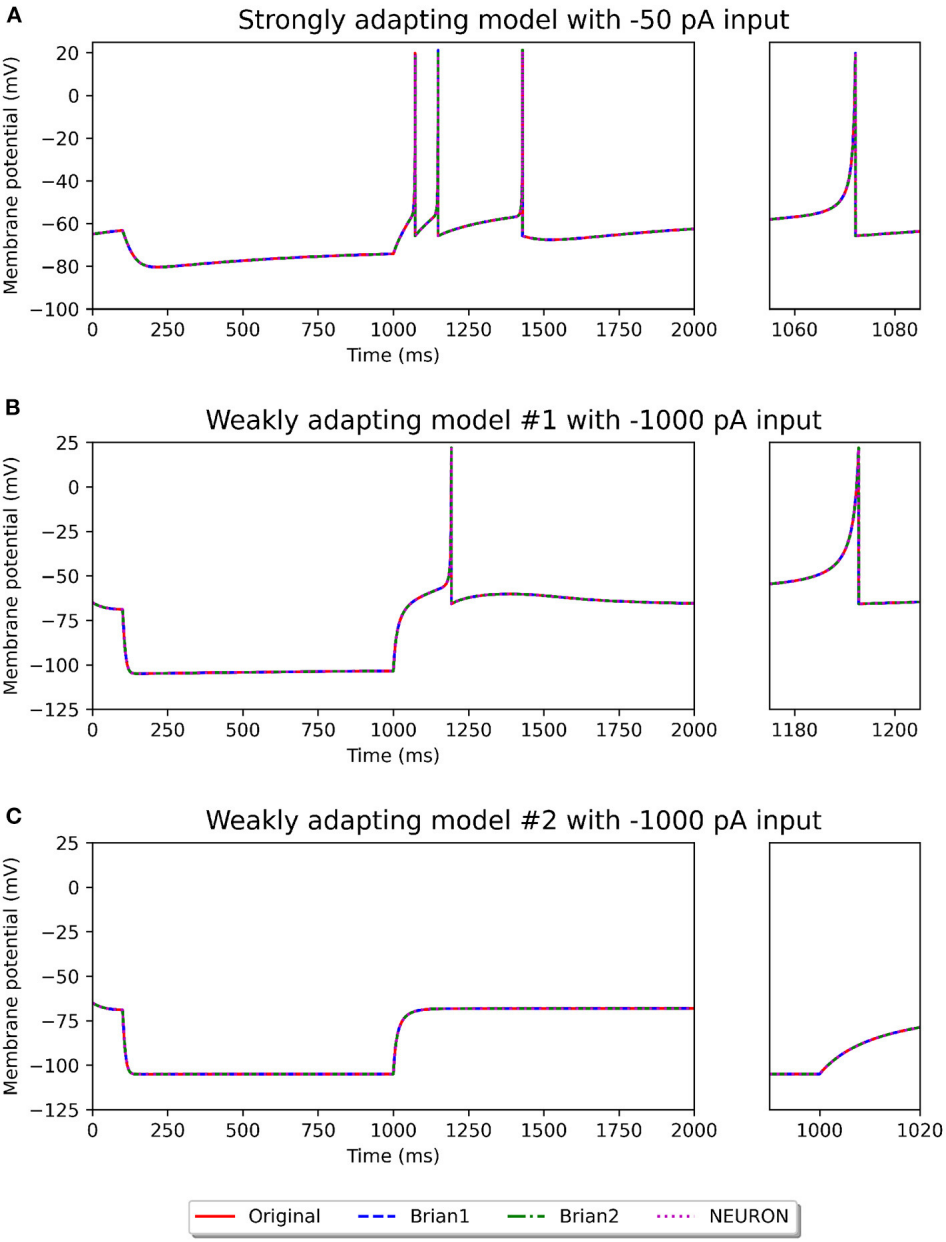
Recording of membrane potential in the different model variants following a hyperpolarizing stimulus. The stimulus strengths were chosen based on the examples in the original study: −50 pA stimulus for the strongly adapting models **(A)**, and −1,000 pA stimulus for the weakly adapting models **(B,C)**. Rebound spiking is observed in the *Pyr_Strong* and *Pyr_Weak1* models, but not in the *Pyr_Weak2* models. The smaller panels on the right provide a closer view of part of the recording.

**Figure 4:**
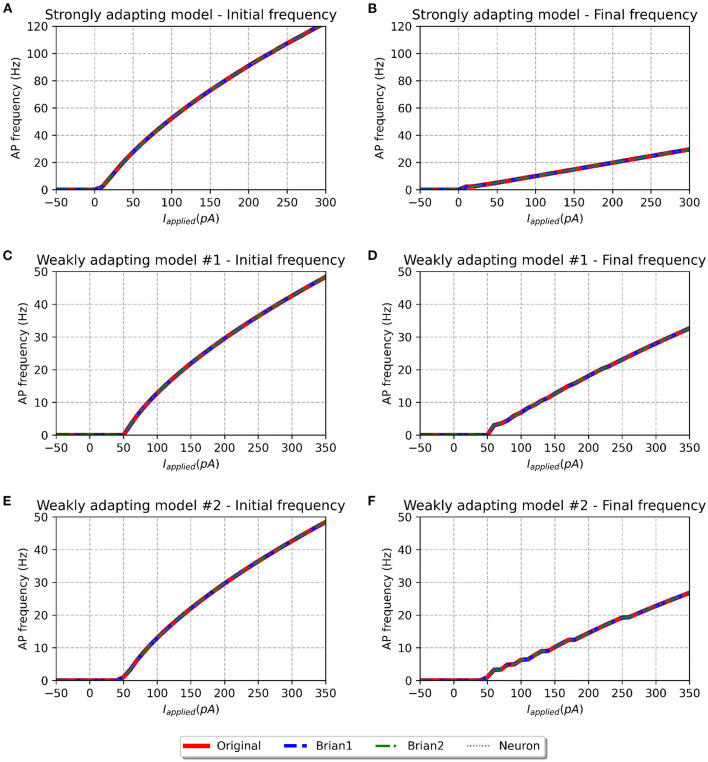
f-I curve for the various models when run with initial membrane potential set to −65 mV. Panels on the left show the initial f-I curve, while those on the right show the final f-I curve. The variation in spiking frequency, difference between initial and final f-I curves, is very noticeable for the strongly adapting models, and comparatively much lower for the weakly adapting cells. All the model implementations show near-identical responses.

Figures 2, 3 show the response of the various models to a stimulus. The stimuli were the same as those employed in the original study (see Figures 4, **6** of original study). In response to a depolarizing stimulus (188 pA for the *Pyr*_*Strong* models, and 154 pA for the *Pyr*_*Weak1* and *Pyr*_*Weak2* models, for a duration of 1 s), all the models exhibit spiking activity. The frequency of these spikes is found to decrease, by different extents, over the course of the stimulation, thereby displaying varying levels of adaptation. All the model implementations are seen to exhibit very similar responses, with only the NEURON implementation showing miniscule differences (see magenta colored dotted line in Figure 2A). Such minor variations between simulators are common and typically arise from differences in the integration methods they adopt internally and/or from the order of operations at every time step. In response to a hyperporlarizing stimulus, the *Pyr*_*Strong* and *Pyr*_*Weak1* models exhibit rebound spiking, wherein cessation of the hyperpolarizing stimulus elicits a spike. This was not observed in the *Pyr*_*Weak2* models for stimuli in the physiological range. These findings align closely to those reported in the published study.

We simulated all the models to obtain their f-I curves. The range of stimuli was the same as in the original study, i.e., between −50 and 300 pA for the strongly adapting models, and −50 and 350 pA for the weakly adapting models. We employed steps of 10 pA in each case. Figure 4 shows the f-I curves for the various models employed in this study. It can be observed that all the implementations of the three model variants (*Pyr*_*Strong*, *Pyr*_*Weak1*, and *Pyr*_*Weak2*) show near-identical responses in comparison to the published model. As expected, the strongly adapting models show a large adaptation in spiking frequencies (e.g., *Pyr*_*Strong* has an initial frequency of ~107 Hz vs. final frequency of ~25 Hz for a stimulus of 250 pA), while the weakly adapting models show much lower adaptation (e.g., *Pyr*_*Weak1* produced an initial frequency of ~48 Hz and a final frequency of ~33 Hz for a 350 pA stimulus; *Pyr*_*Weak2* produced an initial frequency of ~48 Hz and a final frequency of ~27 Hz for a 350 pA stimulus). Also, note that the initial and final frequencies diverge more strongly with increasing strengths of applied stimulus.

Table 2 presents the root mean squared error (RMSE) values as a measure of the goodness-of-fit between the response of the *Original* model, and the other implementations. The perfect scores (RMSE = 0.0) for the *Brian1* set of models confirm that the re-implementation related changes have not introduced any changes in the exhibited biophysical responses. *Brian2* models were also found to exhibit identical responses, with RMSE scores of 0.0. *NEURON* models showed very minor differences.

**Table 2:** Root mean squared error (RMSE) values quantifying the match between the published model and the reproductions produced in this study.

**Parameter**	**Pyr_Strong**				**Pyr_Weak1**				**Pyr_Weak2**			
	**O**	**B1**	**B2**	**N**	**O**	**B1**	**B2**	**N**	**O**	**B1**	**B2**	**N**
*initial_fi (Hz)*	0.00	0.00	0.00	0.12	0.00	0.00	0.00	0.02	0.00	0.00	0.00	0.02
*final_fi (Hz)*	0.00	0.00	0.00	0.01	0.00	0.00	0.00	0.06	0.00	0.00	0.00	0.01

Units of the RMSE values are the same as those of the parameters. O, *Original*; B1, *Brian1*; B2, *Brian2*; N, *NEURON*. The features were evaluated over a range of stimulus strengths. The response of the *Original* model was used as target reference data.

The f-I curve trend for the strongly adapting models shown in Figure 4 matches well with Figure 3 in the original study. Digitizing the original figure shows, for a stimulus of 250 pA, an initial frequency of ~108 Hz and a final frequency of ~25 Hz. This corresponds well with the values evaluated for our reproductions, as reported previously. Also, the authors reported a rheobase of ~0 pA for the *Pyr*_*Strong* model, and our models report a value of 3 pA for the same (evaluated using steps of 1 pA; not shown here), suggesting a fair match.

**Figure 5:**
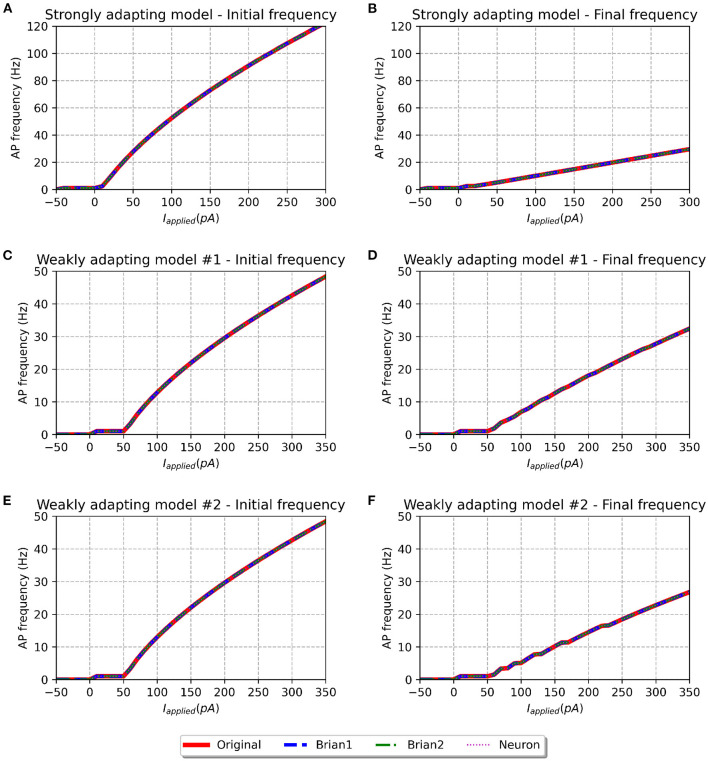
f-I curve for the various models when run with initial membrane potential = −55 mV. Panels on the left show the initial f-I curve, while those on the right show the final f-I curve. It can be observed that the rheobase current is reduced as compared to f-I curves with initial potential of −65 mV.

However, certain notable discrepancies are found for the weakly adapting models, when compared to the findings of the published article (see Figure 5 of the original study). Their figure shows, for a stimulus of 350 pA, *Pyr*_*Weak1* has an initial frequency of ~49 Hz and a final frequency of ~29 Hz (cf. 33 Hz in our models), while *Pyr*_*Weak2* has an initial frequency of ~49 Hz and a final frequency of ~14 Hz (cf. 27 Hz in our models). It is found that the initial frequencies are quite similar for the reproductions, but notable differences are observed for the final frequencies. Our *Pyr*_*Weak2* model, in particular, shows significantly lower adaptation than that in the published study. Further, significant differences are found for the value of rheobase current. The authors report a value of 5 pA for both the weakly adapting models, but our simulations indicate the rheobase current as 51 and 49 pA for the *Pyr*_*Weak1* and *Pyr*_*Weak2* models, respectively. It is important to note that these differences are found not only in the new model implementations, but also in our simulations using the published source code (i.e., the *Original* model).

To investigate if the initial membrane potential adopted in our simulations (−65.0 mV) had any significant role in the observed f-I curve discrepancies, we set the same to −55.0 mV, corresponding to the upper-limit in the published source code. The corresponding f-I curves are shown in Figure 5. All model variants continue to show similar initial and final frequencies for larger stimulus strengths, as reported for an initial membrane potential of −65.0 mV. But differences are observed at smaller stimulus strengths. Though the rheobase of the weakly adapting models (1 pA) is now closer to that published in the study (5 pA), there exists now a large discrepancy in the rheobase of the strongly adapting cell (−44 pA; cf. ~0 pA in published study). Therefore, the change in the initial membrane potential from −65.0 to −55.0 mV appears to have largely influenced only the rheobase currents, without producing any corrective effect on the discrepancies reported in the final spiking frequencies of the weakly adapting models.

### 3.2. Validations: Level II

The perfect match between the *Original* and *Brian1* set of models, as seen in Figure 4 and Table 2 gives confidence that they can be treated as equivalents. As described earlier, the *Brian1* models are SciUnit-compatible, and this enables them to easily undergo the additional validations we have developed. We shall thus employ the *Brian1* set of models as representative of the published model, and use its responses to compare the *Brian2* and *NEURON* sets of models.

A number of electrophysiological features were evaluated using the eFEL library *via* the *eFELunit* test suite that we developed as part of this study. Similar to level I validations, the degree of fit was quantified with the RMSE values. These are listed in Table 3 for the various features that were tested. The stimulus was initiated at the start of the simulation (*t* = 0 ms) and applied for a duration of 1,000 ms. Only when determining *iv*_*curve*, the stimulus was applied after 1,500 ms for a duration of 2,500 ms. This was deemed necessary to ensure that a stable membrane potential could be identified, both before and at the end of the stimulus, in order to accurately evaluate the voltage deflection.

**Table 3:** Root mean squared error (RMSE) values quantifying the match between the reproduced models for various electrophysiological features.

**Parameter**	**Pyr_Strong**			**Pyr_Weak1**			**Pyr_Weak2**		
	**B1**	**B2**	**N**	**B1**	**B2**	**N**	**B1**	**B2**	**N**
*spikecount (no units)*	0.00	0.00	0.00	0.00	0.00	0.00	0.00	0.00	0.00
*time_to_first_spike (ms)*	0.00	0.00	0.05	0.00	0.00	0.06	0.00	0.00	0.07
*time_to_second_spike (ms)*	0.00	0.00	0.09	0.00	0.00	0.07	0.00	0.00	0.09
*time_to_last_spike (ms)*	0.00	0.00	0.31	0.00	0.00	0.17	0.00	0.00	0.14
*AP1_amp (mV)*	0.00	0.00	2.20	0.00	0.00	1.24	0.00	0.00	1.24
*AP2_amp (mV)*	0.00	0.00	2.37	0.00	0.00	0.99	0.00	0.00	1.23
*APlast_amp (mV)*	0.00	0.00	2.99	0.00	0.00	0.60	0.00	0.00	1.08
*AP1_width (ms)*	0.00	0.03	0.05	0.00	0.03	0.07	0.00	0.02	0.05
*AP2_width (ms)*	0.00	0.02	0.05	0.00	0.02	0.05	0.00	0.02	0.05
*APlast_width (ms)*	0.00	0.02	0.05	0.00	0.03	0.05	0.00	0.03	0.06
*iv_curve (MΩ)*	0.00	0.00	0.00	0.00	0.00	0.00	0.00	0.00	0.00

Units of the RMSE values are same as those of the parameters. B1, *Brian1*; B2, *Brian2*; N, *NEURON*. The features were evaluated over a range of stimulus strengths. The response of the *Brian1* model was used as the target reference data.

The perfect match (RMSE = 0.0) for the *spikecount* feature indicates that all the models produced the exact same number of spikes during the course of the simulation, for all values of applied stimulus. In general, it is found that the *Brian1* and *Brian2* models demonstrated near-identical responses, with the RMSE scores ≤ 0.03 for each test.

We next compared the time taken to spike, from the onset of stimulus, and evaluated this for the first, second, and last spikes in the recordings. The *NEURON* models' spike times varied very slightly from the other implementations. This discrepancy is initially small, e.g., at the first spike, but progressively increases and results in a large divergence when comparing the final spike times. This is reflected in the increasing RMSE scores for *NEURON* models when evaluating the timings for the first, second, and last spike. *Brian2* models demonstrated a perfect match, with all the evaluated spike timings identical to those of *Brian1*. It would be relevant to mention here that, in contrast to the Brian simulator, Brian2 simulator, as default, records the values at the beginning of a time-step. This would have introduced a difference of one time-step in the spike-timings, and was avoided by explicitly instructing Brian2 simulator to record the values at the end of the time-step (by setting the *StateMonitor* with when=“end”).

Next, we explored features associated with the spike shapes, namely, the spike amplitude and the half-width of the spike. It was observed that the *Brian1* and *Brian2* models elicited similarly shaped spikes, with identical amplitudes, and very little differences in the spike-widths. *NEURON* models exhibited some discrepancies with regards to the spike amplitudes, while exhibiting very similar spike-widths.

**Figure 6:**
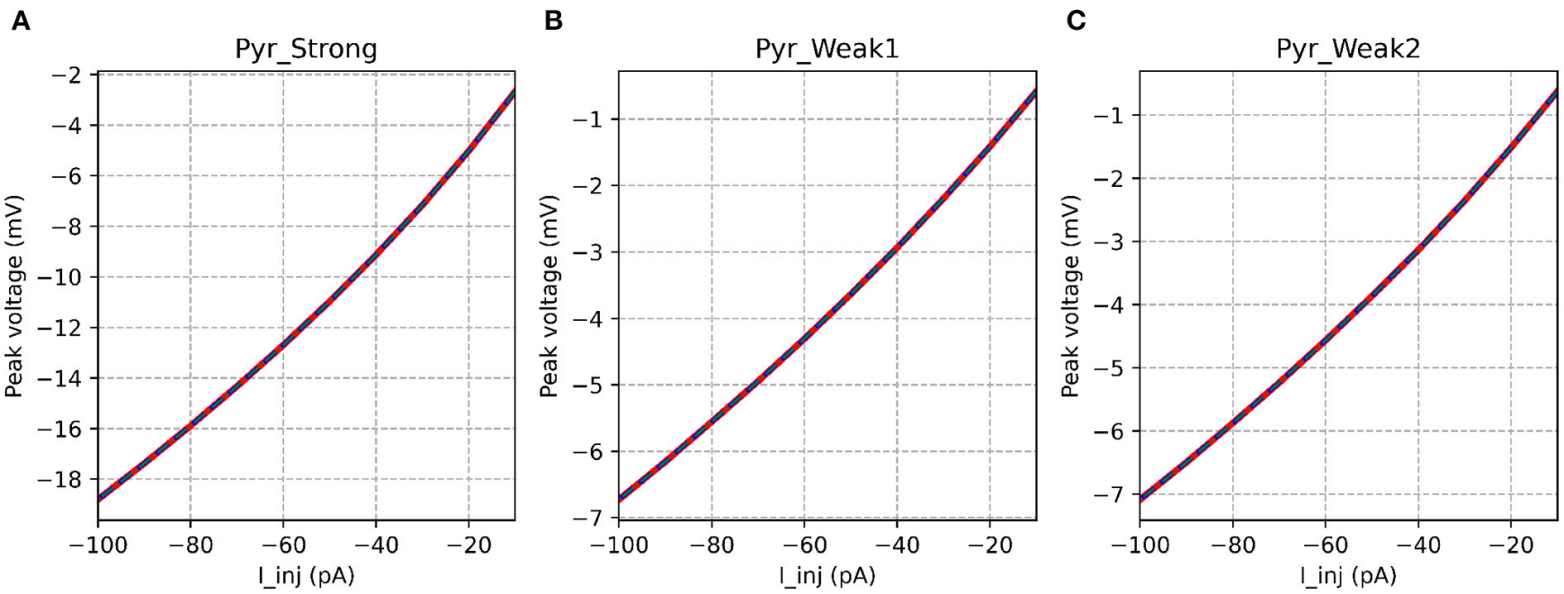
I-V curves for the various implementations. All the implementations show identical response for all model variants. It is evident that the strongly adapting cell model has much higher excitability than the weakly adapting cell models.

Table 3 shows that the models showed a perfect match for the their responses in the passive range, characterized *via* their I-V curves for hyperpolarizing stimuli. This is illustrated in Figure 6.

These tests also allowed us to measure the execution time for simulating the models on the various simulators. As the I-V curve tests required the longest simulations (5,000 ms for each level of stimulus), let us first discuss the models' performances in its context. For the *Pyr*_*Strong* model, Brian1 required 223.45 ms for completing the simulations, while Brian2 and NEURON only required 40.02 and 23.21 ms, respectively. This evaluates to a speed-up of ~5.6x for Brian2 over Brian1, and ~9.6x for NEURON over Brian1. It is interesting to note that for the shorter simulations, for example, when running the test for *spikecount* (1,000 ms for each level of stimulus), the *Pyr*_*Strong* model required 47.14, 8.20, and 2.88 ms, on Brian1, Brian2, and NEURON, respectively. This corresponds to a speed-up of ~5.7x and ~16.4x for Brian2 and NEURON, over Brian1. This suggests that simulations are significantly faster using the NEURON simulator for both shorter and longer sets of simulations. It is pertinent to mention that both NEURON and *Brian2* offer methods to optimize the execution of simulations, which can potentially lead to faster executions. Here, we have only evaluated the out-of-the-box performance for all simulators, without focusing on such optimization aspects.

## 4. Discussion

We have reproduced the simplified CA1 pyramidal cell model, developed by Ferguson et al. (2014) with the Brian2 and NEURON simulators. These implementations are able to reproduce the core features of the original model developed with Brian, but do not completely correspond to that reported in the published study. The original publication provides sufficient information for reproducing the model, but lacks some details with regards to the protocols followed for running the simulations. The availability of the original model, *via* ModelDB, is useful for perusing the source code, but does not alleviate the issues encountered in reproducing the simulation outcomes. Similar differences were found in the simulation outcomes when using the published model, from ModelDB, to reproduce the data in the published study. The differences in the f-I relationship and the rheobase currents of both the weakly adapting pyramidal models are the major discrepancy. Our implementations shows a rheobase current of ~50 pA, while the original study reports this as 5 pA. Also, the extent of adaptation seems to differ. Our weakly adapting models demonstrate much lower adaptation than those in the published study. The source of these discrepancies is not properly identified. One of the possibilities was the value of the initial potential of the neuron at the start of the simulation.

**Figure 7:**
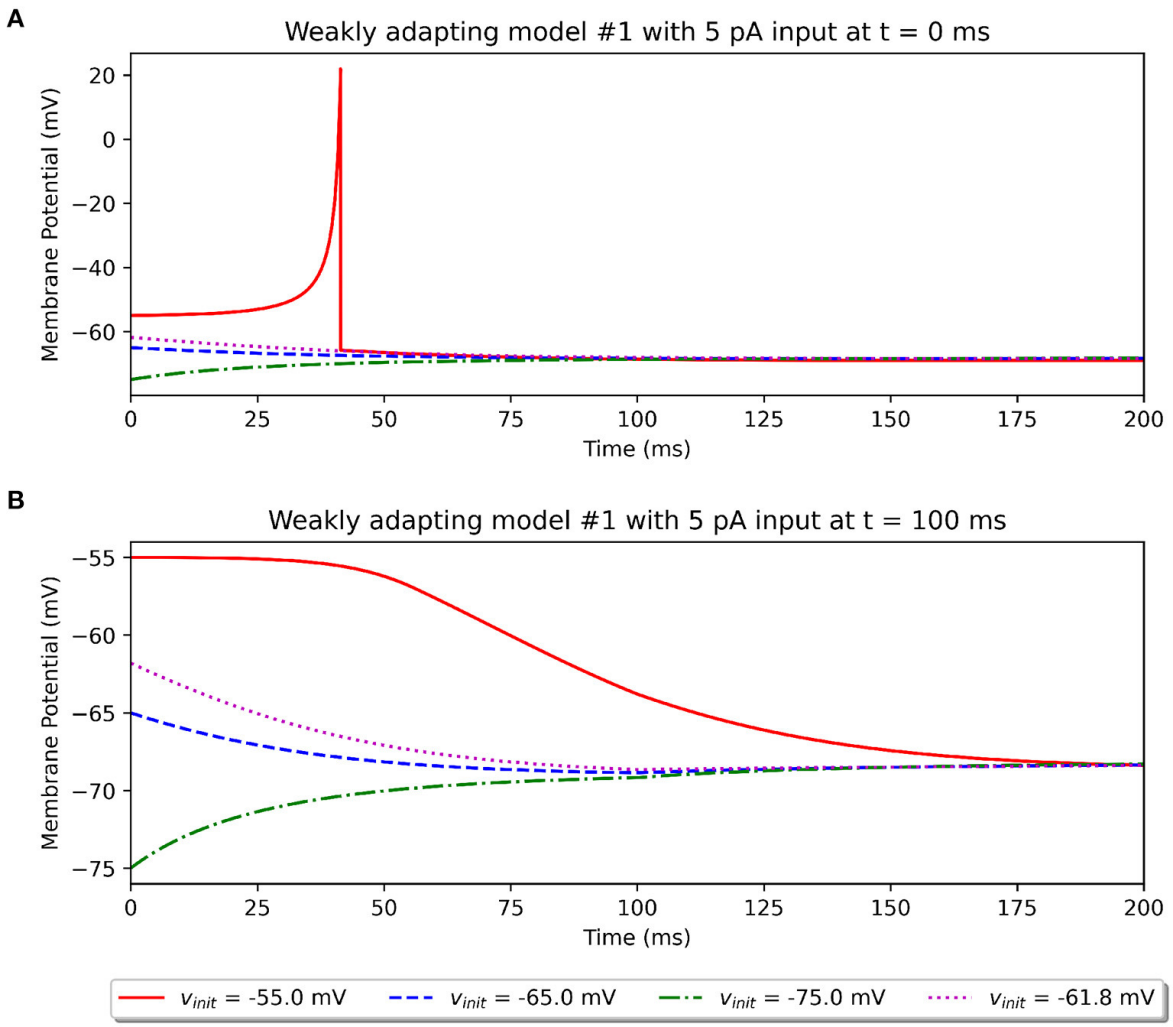
Simulations illustrating the effect of varying values for the initial resting potential using the *Original* model. **(A)** Shows the response to a 5 pA stimulus applied at *t* = 0 ms, and **(B)** shows the same stimulus applied at *t* = 100 ms.

In an attempt to explore the effect of the initial potential on the simulation outcome, we recorded the model response to a 5 pA stimulus (rheobase reported in study) under different initial potentials. As described previously, the published study does not make a mention of this parameter. The model available on ModelDB randomly sets this between −55.0 and −75.0 mV. We explored the effect of setting different initial resting potentials. This is illustrated in Figure 7 for the *Pyr*_*Weak1* model. We tested initial potentials of −55.0, −65.0, and −75.0 mV, corresponding to the minimum, mean, and maximum of the specified range. We also tested for the resting membrane potential value of −61.8 mV, corresponding to the parameter *V*_r_ in Equation (1). It was found that only when the model started from a much depolarized initial potential, was it able to elicit a spike for the 5 pA stimulus, if applied at the start of the simulation (see Figure 7A). If applied after a delay, say of 100 ms, then the membrane would already have equilibrated to a more hyperpolarized potential, and the 5 pA stimulus would no longer suffice to elicit a spike (see Figure 7B). None of the other initial potentials we tested led to a spike.

The above observations demonstrate that two potentially important missing pieces of information are the initial resting potential of the models and the delay (if any) before the stimulus was applied. Our investigations, such as that illustrated in Figure 5, indicate that changing the initial potential to −55 mV would not provide a better match to the published outcomes. Importantly, it should be noted that this choice of initial resting potential and delay before application of stimulus does not in any way affect the comparisons between the various model implementations presented here, as all of them are simulated with identical protocols.

A peculiar trend can be observed in Figure 5 of the original study, for the curve representing the initial frequency of the weakly adapting cells. At around ~55 pA, a kink can be observed, suggesting that no spikes were recorded for that level of stimulus, despite spikes having been recorded for smaller stimuli. Also, the curve for the final frequency at the same stimulus strength indicates the recording of a single spike (1 Hz). The source of this anomaly is hard to identify but could potentially originate from a variable initial potential being employed across the multiple simulation runs. If so, this would further highlight the need for maintaining a constant initial potential across all simulations when characterizing a model.

Another possible explanation for the discrepancy between the published findings and our attempts at replicating them using the *Original* set of models could be a difference in the version of the Brian simulator employed in the original study. Their publication does not provide information about the specific version used. We used the final release (further development has been discontinued) of the Brian simulator, v1.4.4, for running the *Original* and *Brian1* models. The minor syntactical changes that were required to be able to run the published model give weight to this argument. It should also be borne in mind that the model's source code available on ModelDB could potentially be a sample implementation of their model, which might not suffice to reproduce all the findings in their published article. The reproduced models presented here were developed based on the information available in the published article, with only the initial resting potential and the time-step for simulation being borrowed from the ModelDB source code.

On the positive side, our weakly adapting models exhibit even weaker adaptation than that reported in the original study, conforming better to the experimental data that they reported (see Figure 5 of the original study). Overall, the first level of validations indicates very minor differences between the implementations and the published source code, and also gives confidence in using the *Brian1* model as representative of the published model.

We would like to highlight that the purpose of using the output of the *Original* model as target reference data, was with the objective of validating the model reproductions. As the published source code, available *via* ModelDB, itself could not reproduce the published outcomes accurately, there was not much value in using the digitized traces from the published figures as reference data. If used, all the simulated outcomes would fare poorly, including those using the published model, and provide no appraisal of the reproduction efforts. Instead, we used the description of the original model and the available source code to simulate and produce the reference data.

It is likely that some simulation-related details are missing from the descriptions provided in the published article, and incorporating these could possibly help to better align our simulation outcomes to those in the original study. We found some differences in the models, specifically the *NEURON* set of models, when evaluating the spike shapes, such as their amplitudes. But it should be noted that such simplified point-neuron models are typically targeted toward matching physiologically observed spiking activity in terms of their frequencies and timings, with the spike shape often not having much importance. This is also evident in the original study, where the simulated spike shapes are noticeably different from those observed experimentally (see Figure 4A of the published study). These features have been, in part, evaluated here to highlight the variety of tests that are available in the *eFELunit* test suite, and can be employed for both point-neuron models as well as spatially-distributed models.

It is also interesting to note the marked differences in input resistance between the *Pyr*_*Strong* model (224.5 MΩ), and the weakly adapting models (*Pyr*_*Weak1*: 80.5 MΩ, *Pyr*_*Weak1*: 86.5 MΩ); values evaluated between −10 and −30 mV. It would be useful to verify if this difference of ~3x is observed in experimental recordings from such cells.

For the *NEURON* models, we had initially employed the *derivimplicit* integration method (results not presented here; available *via* the associated EBRAINS Live Paper) and later switched to the *euler* method. The latter corresponded to the approach adopted in the original study using the Brian simulator. This switch was found to produce results that, in general, were better aligned with the *Original* models. It is therefore useful to note that such simulator specific choices could also affect model comparisons.

In light of the replication study undertaken here, it would be opportune to propose certain good practices that could be adopted when reporting computational modeling studies. These include: (i) uploading the model to a well-established online repository (e.g., ModelDB, GitHub) that would ensure their long term availability, (ii) tabulating values of the various parameters employed in the study, and clearly state in text if any of these have been altered for certain simulations, (iii) describing the mathematical equations, if any, that were implemented in simulator specific syntax, (iv) providing detailed steps or scripts to reproduce findings reported in the article, (v) documenting the versions of packages employed, especially that of the simulators, (vi) providing steps to create and setup the simulation environment from scratch. (vii) specifying any simulator specific parameters that are necessary to reproduce the findings accurately (e.g., the time step used for simulations, the numerical integration method employed).

The above listing is not intended to be exhaustive, but attempts to lay down basic steps that can assist with reproducibility and replication. Very often details associated with model development are clearly presented in the article but lack sufficient insight into the simulation protocols. For example, it is common to indicate the strength of the applied stimulus, but to omit the time at which it was applied, and the duration for which it was active; these often have to be estimated based on any relevant figures. Some of the information proposed above could seem excessive to be included within the article, owing to considerations such as the length of the article. It is recommended to present these *via* supplementary documents tied to the publication. The EBRAINS Live Paper employed for this article presents one such approach. Many of these issues can be addressed by providing the source code necessary for developing the model and for running the simulations.

All resources that were created and/or employed in this study have been publicly shared through a Live Paper. This provides readers with access to all the data and simulation artifacts, along with integrated tools for exploring them. Additionally, all models and tests discussed in this study have been registered in the EBRAINS Model Catalog, thereby providing access to additional relevant metadata. All validation results, along with data files and figures produced as part of the evaluation, are linked to the models and tests, and publicly available online. These measures greatly help in satisfying the Findable (F), Accessible (A), and Reusable (R) aspects of the FAIR principles for sharing scientific data (Poline et al., 2022).

In the future, we plan to extend the *eFELunit* test suite to accommodate all features that can be extracted using the eFEL library. This would provide a SciUnit compatible approach that would readily enable such models to evaluate a multitude of features. Additional flexibility would be incorporated, such as to allow comparisons over diverse stimuli, and also a choice of additional methods to quantify the goodness of fit, such as *via* R-squared, mean absolute error (MEA) and normalized root mean squared error (NRMSE).

Through this study, we hope to have demonstrated an approach to validating the reproducibility of published models, not just through a simple visual assessment of likeness, but through quantitative evaluation of similarity across a multitude of biophysical parameters. The method adopted here, using the SciUnit framework, offers a generalized approach that can be easily employed in other replication and reproduction studies.

## Data availability statement

All resources employed and generated in this study are publicly accessible through the corresponding EBRAINS Live Paper located at: https://live-papers.brainsimulation.eu/#2022-appukuttan-davison. Resources are also available on our GitHub repository: https://github.com/appukuttan-shailesh/Ferguson2014_Repro.

## Author contributions

SA designed and executed the study and wrote the first draft of the manuscript. AD helped design the study and revised the manuscript. All authors have reviewed and approved the manuscript.

## Funding

This project was developed in part or in whole in the Human Brain Project, funded from the European Union's Horizon 2020 Framework Programme for Research and Innovation under Specific Grant Agreement No. 945539 (Human Brain Project SGA3).

## Conflict of interest

The authors declare that the research was conducted in the absence of any commercial or financial relationships that could be construed as a potential conflict of interest.

## Publisher's note

All claims expressed in this article are solely those of the authors and do not necessarily represent those of their affiliated organizations, or those of the publisher, the editors and the reviewers. Any product that may be evaluated in this article, or claim that may be made by its manufacturer, is not guaranteed or endorsed by the publisher.
